# Intimal Hyperplasia in Balloon Dilated Coronary Arteries is Reduced by Local Delivery of the NO Donor, SIN-1 Via a cGMP-Dependent Pathway

**DOI:** 10.1186/1471-2261-11-30

**Published:** 2011-06-11

**Authors:** Jan Harnek, Evita Zoucas, Valéria Perez de Sá, Eva Ekblad, Anders Arner, Unne Stenram

**Affiliations:** 1 Department of Coronary Heart Disease. Skane University Hospital. Institute of Clinical Sciences. Lunds University, Getingev 4, SE-22185 Lund, Sweden; 2 Department of Surgery. Skane University Hospital. Institute of Clinical Sciences. Lunds University. Getingev 4, SE-22185 Lund, Sweden; 3 Department of Anaesthesiology. Skane University Hospital. Institute of Clinical Sciences. Lunds University, Getingev 4, SE-22185 Lund, Sweden; 4 Department of Experimental Medical Science. Skane University Hospital. Institute of Clinical Sciences Lunds University, Getingev 4, SE-22185 Lund, Sweden; 5 Department of Cellular Musclephysiology. Institute of Physiology and Pharmacology, Karolinska Institute, 171 77 Stockholm, Sweden; 6 Department of Pathology. Skane University Hospital. Institute of Clinical Sciences. Lunds University. Getingev 4, SE-22185 Lund, Sweden

**Keywords:** Nitric oxide, Angioplasty, Endothelium-derived factors, Restenosis, Remodeling

## Abstract

**Background:**

To elucidate the mechanism by which local delivery of 3-morpholino-sydnonimine (SIN-1) affects intimal hyperplasia after percutaneous transluminal coronary angioplasty (PTCA).

**Methods:**

Porcine coronary arteries were treated with PTCA and immediately afterwards locally treated for 5 minutes, with a selective cytosolic guanylate cyclase inhibitor, 1 H-(1,2,4)oxadiazole(4,3-alpha)quinoxaline-1-one (ODQ) + SIN-1 or only SIN-1 using a drug delivery-balloon. Arteries were angiographically depicted, morphologically evaluated and analyzed after one and eight weeks for actin, myosin and intermediate filaments (IF) and nitric oxide synthase (NOS) contents.

**Results:**

Luminal diameter after PCI in arteries treated with SIN-1 alone and corrected for age-growth was significantly larger as compared to ODQ + SIN-1 or to controls (p < 0.01). IF/actin ratio after one week in SIN-1 treated segments was not different compared to untreated segments, but was significantly reduced compared to ODQ + SIN-1 treated vessels (p < 0.05). Expression of endothelial NADPH diaphorase activity was significantly lower in untreated segments and in SIN-1 treated segments compared to controls and SIN-1 + ODQ treated arteries (p < 0.01). Restenosis index (p < 0.01) and intimal hyperplasia (p < 0.01) were significantly reduced while the residual lumen was increased (p < 0.01) in SIN-1 segments compared to controls and ODQ + SIN-1 treated vessels.

**Conclusions:**

After PTCA local delivery of high concentrations of the NO donor SIN-1 for 5 minutes inhibited injury induced neointimal hyperplasia. This favorable effect was abolished by inhibition of guanylyl cyclase indicating mediation of a cyclic guanosine 3',5'-monophosphate (cGMP)-dependent pathway. The momentary events at the time of injury play crucial role in the ensuring development of intimal hyperplasia.

## Background

Endothelial injury after PTCA results in denudation or even rupture of the internal elastic lamina, causing damage to smooth muscle cells (SMC) and release of signal substances, which in turn contribute to neointimal formation and often restenosis [1].

Endothelial-derived NO reduces these events and modulates several physiological processes in the vasculature, including vascular tone, SMC migration, leukocyte adhesion, platelet adhesion and aggregation [2]. The biologic response of SMC and endothelial cells to NO differs. NO inhibits SMC proliferation and can increase apoptotic SMC cell death [3]. On the contrary NO in physiological concentrations enhances endothelial proliferation and reduces endothelial cell apoptosis [4-7]. NO exerts its effects both by a cGMP-dependent and a cGMP-independent pathway [8].

Orally or intravenously administered NO precursors and donors have been shown to moderately affect intimal hyperplasia after arterial injury in animal and human studies [9-12]. These studies have been hampered by the fast degradation rate of NO in blood, which allows only minimal amounts of NO, if any, to reach the injured area. However, intrapericardial delivery of NO has been shown to inhibit PTCA induced restenosis [13]. The NO donor SIN-1 has recently been shown to reduce intimal hyperplasia after PTCA, and may be advantageous as it releases NO by fast and spontaneous degradation[14].

The aim of the present study was to evaluate the effect of locally delivered NO on intimal hyperplasia after PTCA, and to investigate whether the NO donor SIN-1 affects intimal hyperplasia via a cGMP dependent pathway.

## Methods

Animal care and handing followed guidelines in the National Institutes of Health (NIH publication No 85-23, revised 1996). The local ethical committee for animal care approved the study. Twenty-nine domestic pigs weighing 21.5 ± 0.6 kg were premedicated with azaperone 2 mg/kg intramuscularly 30 minutes before the procedure. After induction of anesthesia with thiopental 5-25 mg/kg the animals were orally intubated with cuffed endotracheal tubes. A slow infusion of 1.25 μl/mL Fentanyl in Ringer-acetate was started at a rate of 1.5 mL/min and adjusted as needed. Mechanical ventilation was then established with a Siemens-Elema 300 B ventilator with a mixture of nitrous oxide (70%) and oxygen (30%). Hypnosis was complemented with small intermittent doses of 5 mg meprobamat as needed.

An 8 F introducer sheath (Cordis, USA) was inserted into the left carotid artery and 10,000 IU Heparin given. After an angiogram, PTCA was performed in the left anterior descending artery (LAD) and in the left circumflex artery (LCX). The artery was dilated at 8 atm with a 20 × 3.0 or 3.5 mm dilation balloon (Boston Scientific, USA) using balloon: artery ratios 1.3:1. Before each balloon inflation the pig was given intra-cardially 100 μg nitro-glycerine to avoid spasm and to obtain consistent epicardial artery dilation. Inflation time was 30 s, and immediately after deflation a corresponding 3 or 3.5 × 20 mm drug delivery catheter (Dispatch, Boston Scientific, USA) was inserted into the PTCA-treated site and inflated at 8 - 10 atm. Effort was taken to minimize the time from balloon angioplasty to the time of drug treatment (approximately 1 minute), in order to avoid thrombus formation. Infusion with 10^-5 ^M ODQ, a selective inhibitor of NO-sensitive guanylyl cyclase activity (Tocris Cookson, UK.) was performed at a rate of 1 ml/min. for 5 minutes. The Dispatch balloon was flushed with 1 ml saline and continued with infusion of 5 ml 10^-4 ^M SIN-1 for 5 minutes. The selected high dose has been used in earlier studies, and we have recently demonstrated an inhibiting effect on intimal hyperplasia after 10 minutes [15-17]. A 0.5-mm over-dilation was performed for 30 seconds in the corresponding, still untreated LAD or LCX, which were thereafter treated only with SIN-1. In five animals arteries used as controls, underwent PTCA and were treated with NaCl 1 ml/min for 10 minutes. Treatment of the arteries was randomized.

During coronary artery occlusion perfusion pressure was increased by a dopamine infusion and small doses of noradrenaline (10 μ g). Xylocain, 10 mg/kg was injected i.v. before releasing the occlusion to prevent the occurrence of malignant arrhythmias during reperfusion.

The catheters were withdrawn after a final angiogram. The left carotid artery was surgically reconstructed, and the skin sutured. Postoperatively, the pigs received 0.15 mg buprenorphine and 5 ml Streptocillin Vet. The pigs were given 250 mg aspirin daily starting at the day of the procedure.

One week after the angioplasty, one group of eleven pigs was sedated and euthanized with an intra-cardiac injection of 40-mmol potassium chloride. The treated segments were harvested immediately, using micro-surgical technique and cut into a proximal, a medial and a distal segment. An untreated segment was also obtained distal to the treated area. The vessel segments were analyzed electrophoretically for the following proteins actin, myosin and intermediate filaments (IF) and histochemically for NADPH diaphorase in order to visualize the presence of NOS.

Eight weeks postoperatively a second group of sixteen pigs was euthanized as described above. Coronary angiograms were performed and the arteries perfusion-fixated at 100 mmHg with 4% formaldehyde before harvesting. The arteries were examined by routine histology and NADPH histochemistry. A perfusion-fixated vessel segment distal to each of the treated areas was harvested as well.

### Angiographic examination

Fluoroscopic video recordings and angiographic stills were examined. Using the guide catheter as reference, luminal diameter was calculated before, immediately after treatment, and at 8 weeks. Pigs grow fast in 8 weeks, in order to determine the growth of the artery; the diameter of the proximal reference segment, approximately 1 cm proximal to the treated area, was measured at the time of treatment and after 8 weeks. Artery growth was thus calculated as the difference in diameter of the arterial reference segments.

The absolute diameter of the treated segments at 8 weeks was reduced by age growth, in order to measure the angiographic late loss without the influence of natural growth in this non-stent model.

### Electrophoretic examination

To investigate the relative content of actin, myosin and intermediate filament protein (IF) in smooth muscle cells (SMC's), the proteins were separated on one-dimensional SDS polyacrylamide gel electrophoresis (SDS-PAGE). The vascular preparations were homogenized in an SDS-buffer (composition: 25 mM tris (hydroxymethyl) aminomethane HCl (pH 6.8), 2% SDS, 5% mercaptoethanol and 10% glycerol) at a concentration of 50 μl/mg tissue wet weight. The homogenate was then boiled for 2 minutes and centrifuged at 10,000 rpm for 5 minutes. The supernatant was removed and stored at -20°C before electrophoretic analysis. The SDS-PAGE was performed essentially as described by Malmqvist et al. [18] using 8% polyacrylamide gels in a BioRad minigel system. The gels were stained with Coomassie blue, destained and scanned using a GS-300 densitometer (Hoeffer Scientific Instruments, USA). The areas under the myosin heavy chains, actin and intermediate filament bands were evaluated. The ratios of actin/myosin and intermediate filaments/actin were calculated from these areas.

### NADPH histochemistry

The vascular segments were fixed overnight in a mixture of 2% formaldehyde and 0.2% picric acid in phosphate buffer (pH 7.2) or in buffered 4% formaldehyde, followed by rinsing in Tyrode's solution containing 10% sucrose. Specimens were frozen on dry ice and cut in a cryostat at a thickness of 10 μm. NADPH diaphorase activity was rendered visible by incubation of the sections for 45 minutes in 0.1 M Tris-HCl (pH 7.2) containing 1 mM NADPH (Sigma, USA), 0.2% Triton X-100 at 37°, followed by washing in Tris-HCl [19].

Each segment was cut into multiple sections, examined under a light microscope and photographed with maximum of light, creating a completely yellow photograph where only NADPH cells stained dark blue. The images obtained were transferred to a CD-ROM in Kodak format and imported into Photoshop 5.5 (Adobe Inc., USA). These photographs were subtracted into a black and white image. Due to the gray scale practically only the NADPH-stained endothelial cells were black. A 45 × 15 mm representative portion of the cross sectional screen-area consisting of endothelium and media was selected. From the histogram menu, the percentage of black pixels in the photo was registered as percentage of the total number of pixels in the selection.

### Histo-morphological examination

Each sample was cut further into 4-14 sections, which were stained with haematoxylin-eosin for routine histological evaluation. A similar number of sections was cut and stained with van Gieson elastica and used for morphological measurements. Measurements were performed without knowledge of the treatment of the segments.

The intimal (I), medial (M), and luminal (L) areas as well as the internal elastic lamina (IEL) circumference and the IEL fracture length (F) were measured using computerized digital planimetry with a video microscope (Olympus BX 50 F4, Japan) and customized software (Analysis 3.0, Soft Imaging System, Germany). Vessel size was assessed by measuring the area circumscribed by the outer border of the external elastic lamina (EEL area). Injury index = F/IEL, was calculated as fracture length of the IEL normalized for the size of the artery by the circumference of the IEL. Intimal hyperplasia was also normalized for the total artery wall area: I/(I+M). The restenosis index = ((I/(I+M))/(F/IEL) could then be calculated taking into account the degree of injury. Changes in vessel geometry after injury and repair denoted the residual lumen and were defined as L/(L+I) [20,21].

### Summary of the study design

A total of 29 pigs were treated with local drug delivery in the LAD as well as the LCX with either SIN-1 alone or ODQ + SIN-1, except for the control pigs were only NaCl was used. After 7 days 11 pigs were sacrificed and the treated segments examined by electrophoresis and NADPH histochemistry. 56 days later the rest were sacrificed, 5 control animals and 11 pigs were examined by NADPH, angiography, electrophoresis, histochemistry, and histo-morphology

### Statistics

One-way ANOVA with Bonferroni post hoc test was used to compare multiple means. Mann-Whitney test was used for comparison between two groups. p values < 0.05 were considered significant. Data are presented as mean ± SD.

## Results

Two pigs, which were excluded from the study, died peri-procedurally due to ischemic events. The remaining pigs survived the procedure without complications. The pigs weighed at baseline 21.5 ± 0.64; after 7 days 23.1 ± 1.28 and increased to 41.5 ± 2.14 Kg at 56 days. Blood pressure was stable throughout the drug delivery procedure.

### Angiographic examination

The diameters of the proximal reference segments, used for calculating age growth, were 3.24 ± 0.40 mm at day 0 and 3.73 ± 0.44 mm at 8 weeks resulting in 0.55 ± 0.31 mm diameter growths (17%). There was no significant difference in growth between treatment groups.

The angiographic results are presented in (Table 1). With or without age correction SIN-1 treated arteries had significantly larger diameter compared to control (p < 0.01). Following ODQ + SIN-1 treatment vascular diameter was not changed significantly compared to control.

**Table 1:** Angiographic characteristics

Diameter measurements	ODQ+ SIN-1 mm n = 11	SIN-1 mm n = 11	NaCl Control mm n = 5	P -value
Pre- treatment (A)	2.62 ± 0.36	2.76 ± 0.35	2.81 ± 0.41	ns

After treatment (B)	2.77 ± 0.38	2.93 ± 0.34	3.10 ± 0.37	ns

After 8 weeks (C)	3.17 ± 0.35	3.53 ± 0.30	3.07 ± 0.25	< 0.01

Age-corrected at 8 weeks (D)	2.60 ± 0.32	3.12 ± 0.41	2.62 ± 0.29	< 0.01

Late Loss (B-D)	0.21 ± 0,30	-0.19 ± 0,51	0.48 ± 0,31	< 0,05

**Legend to table 1: **Age related growth of the arteries was similar for segments treated either with SIN-1 or ODQ + SIN-1 or to controls. With or without age correction, SIN-1 treated arteries had after 8 weeks significantly larger diameter compared to ODQ + SIN-1 or control segments. Data presented as mean ± SD. One way ANOVA test.

Late loss occurring in treated segments after 8 weeks was calculated as the artery diameter after PTCA at day 0 minus the diameter at 8 weeks adjusted for growth.

### Electrophoretic examination

**Figure 1:**
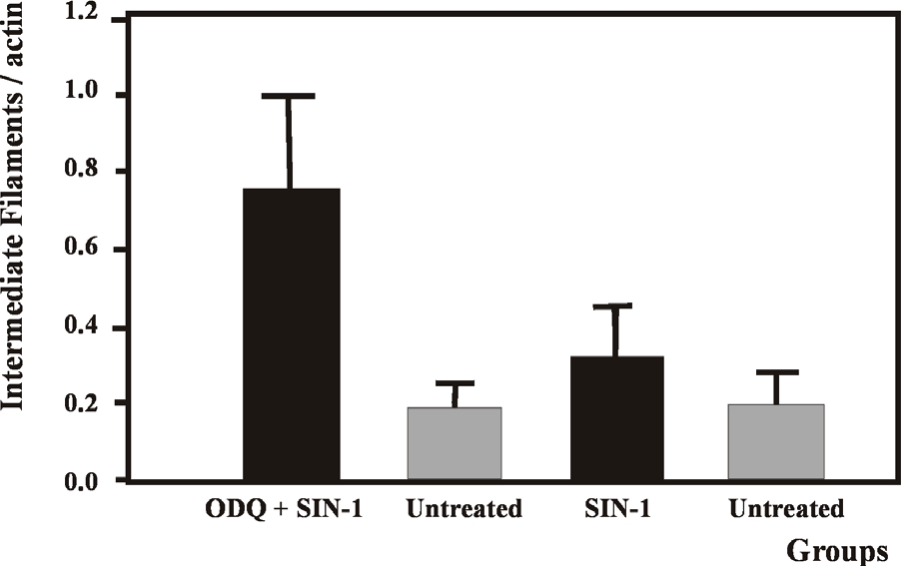
**Artery wall ratio of intermediate filaments/actin after PTCA with ODQ prior to SIN-1 treatment or SIN-1 alone**. **Legend: Figure 1 **depicts the ratio of intermediate filaments to actin in treated and untreated segments. ODQ + SIN-1 caused a significant increase in the ratio compared to untreated segments and segments treated only with SIN-1 (p < 0.05). All groups summarize data from six animals. Data presented as mean ± SD. Mann-Whitney test.

**Figure 2:**
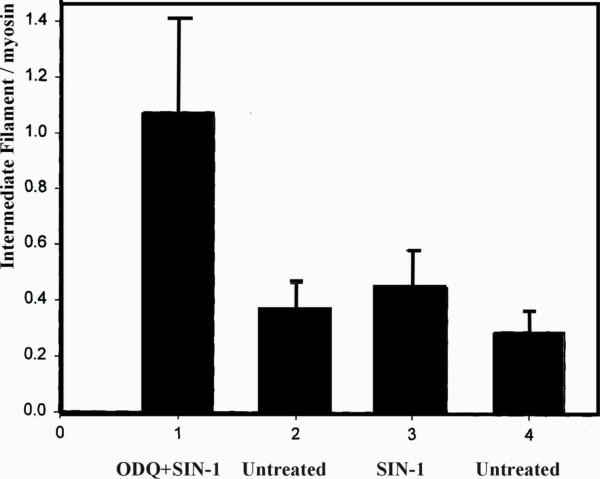
**Artery wall ratio of intermediate filaments/myosin after PTCA with ODQ prior to SIN-1 treatment or SIN-1 alone**. **Legend: Figure 2 **depicts the ratio of intermediate filaments to myosin in treated and untreated segments. ODQ + SIN-1 caused a non-significant increase in the ratio compared to untreated segments and segments treated only with SIN-1 (p = 0.06). All groups summarize data from six animals. Data presented as mean ± SD. Mann-Whitney test.

Biochemical analysis of the effect of SIN-1 on intermediate filament proteins one week after treatment is presented in (Figure 1). The ratio of IF/actin in SIN-1 treated segments did not differ significantly from the corresponding ratios in untreated segments. PTCA followed by ODQ prior to SIN-1 treatment caused a significant increase in the IF/actin ratio compared to untreated arteries as well as SIN-1 treated vessels. IF/myosin ratio was not affected by SIN-1 whereas PTCA followed by ODQ+ SIN-1 treatment non-significantly increased the ratio (p = 0.06) (Figure 2). We could not detect any statistically significant changes in the relative amount of myosin heavy chain normalized to actin.

### NADPH histochemistry

**Figure 3:**
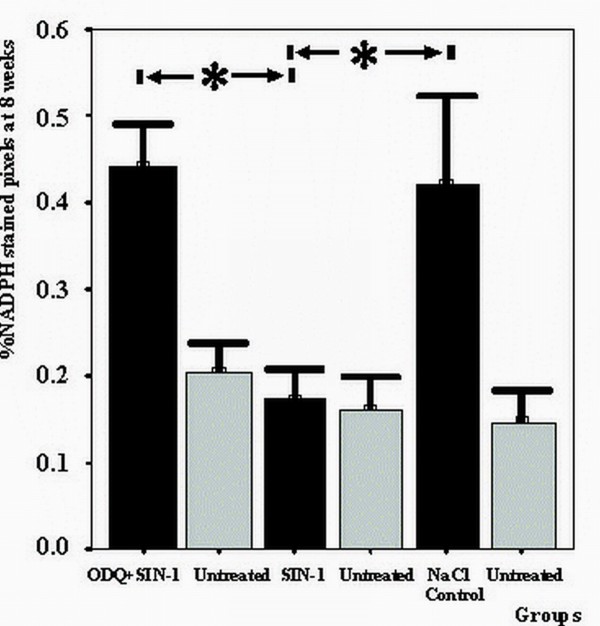
**NADPH stained pixels 8 weeks after PTCA treated with ODQ prior to SIN-1 or SIN-1 alone**. **Legend to figure 3: **NADPH staining at 8 weeks was significantly deceased in SIN-1 treated arteries compared to controls (p < 0.01). There was no significant difference between SIN-1 treated and untreated segments. NADPH staining in control segments and ODQ + SIN-1 treated segments was increased compared to untreated arteries. There was no difference in NADPH staining between controls and ODQ + SIN-1 treated vessels. All groups summarize data from six animals. Data presented as mean ± SD. * indicates p < 0.05 One-way ANOVA with Bonferroni post hoc test.

At 8 weeks saline treated controls showed significant increase in NADPH staining compared to untreated vessels (p < 0.01) as a normal response to artery injury. The increase was inhibited after PTCA with SIN-1treatment, whereas PTCA with ODQ prior to SIN-1 treatment resulted in significant elevation of NADPH staining (p < 0.01) (Figure 3).

**Figure 4:**
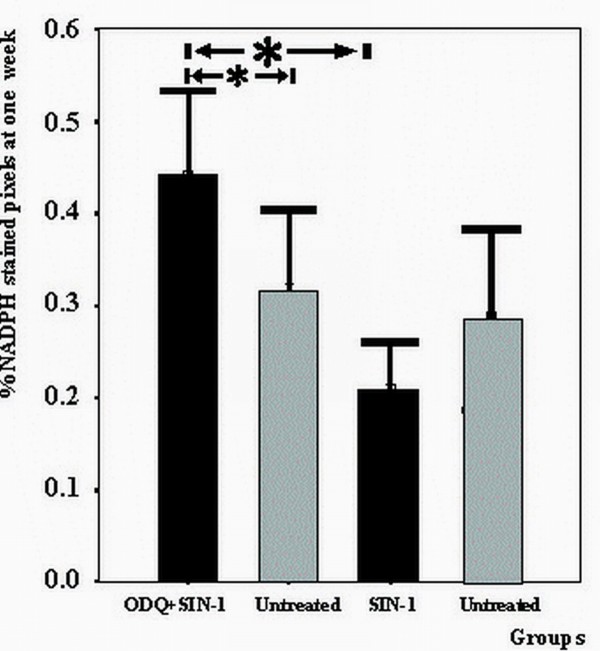
**NADPH stained pixels 1 week after PTCA treated with ODQ prior to SIN-1 or SIN-1 alone**. **Legend to figure 4: **At one week there was no significant difference in NADPH staining between the SIN-1 treated arteries compared to untreated vessels. NADPH staining was significantly higher in ODQ + SIN-1 treated segments compared to SIN-1 treated as well as untreated vessels (p < 0.01). All groups summarize data from six animals. Data presented as mean ± SD. * indicates p < 0.05 Mann-Whitney test.

The increase in NADPH staining after ODQ + SIN-1 application was detectable one week after treatment (Figure 4).

Changes in NADPH diaphorase activity were circumscribed to treated segments while there were no changes observed in correlating matched distal untreated segments.

### Histo-morphological examination

8 weeks after treatment all arteries were patent. There were no statistically significant differences in the internal elastic lamina fracture lengths inflicted to the arteries. In the SIN-1 treated segments, the restenosis index (0.60 ± 0.65) was 72% less and the amount of intimal hyperplasia (0.07 ± 0.06) 71% reduced compared to controls. The residual lumen (0.92 ± 0.08) was 26% larger in SIN-1 segments and there were no differences in EEL compared to controls. Intimal hyperplasia is significantly decreased after SIN-1 treatment compared to controls (p < 0.01). Addition of ODQ prior to SIN-1 treatment resulted in response to injury similar to the response of controls (NaCl) (Table 2).

**Table 2:** Histo-pathological characteristics of PTCA injured coronary arteries 8 weeks after treatment with ODQ + SIN-1 and SIN-1 and control

8 weeks after treatment	ODQ+ SIN-1 n = 11	SIN-1 n = 11	NaCl Controls n = 5	P -value
EEL area (mm^2^)	2.79 ± 0.55	2.64 ± 1.11	3.28 ± 1,16	ns

Medial area(M) (mm**^2^**)	1.16 ± 0.36	1.27 ± 0.46	1.27 ± 0.47	ns

Intimal area (I) (mm**^2^**)	0.50 ± 0.59	0.11 ± 0.10	0.73 ± 0.69	< 0.01

Luminal area (L) (mm**^2^**)	1.12 ± 0.36	1.26 ± 0.67	1.28 ± 0.91	ns

IEL length (mm)	3.91 ± 1.06	4.40 ± 0.73	4.20 ± 1.37	ns

Fracture length (F) (mm)	0.71 ± 0.54	0.91 ± 0.73	1.21 ± 0.77	ns

Injury index**^1^**	0.22 ± 0.21	0.21 ± 0.19	0.35 ± 0.29	ns

Residual lumen**^2^**	0.73 ± 0.26	0.92 ± 0.08	0.63 ± 0.30	< 0.01

Restenosis index**^3^**	2.10 ± 2.24	0.60 ± 0.65	1.02 ± 0.70	< 0.01

Intimal hyperplasia**^4^**	0.25 ± 0.23	0.07 ± 0.06	0.32 ± 0.24	< 0.01

Results are presented as mean ± SD. One-way ANOVA was used for statistical evaluation. EEL: External elastic lamina. IEL: Internal elastic lamina.

^1^Injury index: IEL fracture length/IEL circumference.

^2^Residual lumen: L/(L+I)

^3^Restenosis index: (I/I+M)/(F/IEL)

^4^Intimal hyperplasia: I/(I+M)

**Legend to table 2: **PTCA followed by SIN-1 treatment demonstrated significant larger residual lumen and reduced intimal area, restenosis index, and intimal hyperplasia compared to NaCl treated or if treated with ODQ prior to SIN-1. Data presented as mean ± SD. One way ANOVA test.

## Discussion

This study shows that delivery of the NO donor SIN-1 at the site of a PTCA injury for 5 minutes decreases significantly intimal hyperplasia/restenosis. Inhibition of guanylyl cyclase by ODQ attenuated the beneficial effect of NO delivery indicating the involvement of a cGMP dependent pathway. In this experiment additional ODQ to the delivery system did not increase intimal hyperplasia compared with controls.

Application of a NO donor was chosen in the present study, since NO interferes with the restenotic process at several levels. NO has three major properties in the vascular system: anti-ischemic/anti-hypertensive, anti-atherosclerotic and anti-thrombotic. Nitric oxide interferes with most intracellular messengers because it diffuses rapidly and isotopically through tissues [2].

The action of NO in regulating growth and migration of vascular smooth muscle cells is mainly mediated through a cGMP-dependent mechanism [22,23]. Activated platelets participate by adhering to endothelial ulcerations and releasing mitogens and cytokines, which induce SMC migration and proliferation. SMCs in the vascular lesion change their phenotype from contractile to secretory [8]. In the present study we examined whether the beneficial effect of an externally applied NO donor in a high concentration could be modified by a guanylyl cyclase inhibitor, as an indication for the implication of a cGMP dependent pathway in the development of intimal hyperplasia/restenosis.

NO, derived from endothelial cells, modulates SMC migration and reduces production of extracellular matrix [24,25]. In physiological concentrations NO contributes to human endothelial cell survival, whereas in high concentrations NO induces endothelial cell apoptosis [26,27]. SIN-1 spontaneously releases NO, which can further react with superoxide and at high concentrations form peroxynitrite (ONOO^-^) [28]. NO scavenges superoxide anion directly and independent of cGMP. Peroxynitrite is a selective oxidant [29] causing nitration of structural proteins [28]. SIN-1 in concentrations of 400 μM has been shown to result in necrosis of cell cultures due to peroxynitrite. However, concentrations of 200 μM SIN-1 caused cellular apoptosis [30]. In the present study it is plausible that production of peroxynitrite at a concentration of 100 μM SIN-1, affected our results. However, since the effect of SIN-1 on intimal hyperplasia was inhibited in the presence of ODQ, a cytosolic guanylyl cyclase inhibitor, this assumption seems unlikely.

PTCA treatment causes intimal hyperplasia [20]. In the present study we have shown angiographically increased artery diameter after local delivery of SIN-1. A clinical multicenter study (ACCORD) indentified modest improvement of restenosis after intravenous or oral administration of sydnonimines [11]. Yet, a recent randomized trial of 166 patients failed to show benefit of 8 mg/day controlled release tablets for 6 month on restenosis after PTCA [12]. Site-specific delivery of SIN-1 affects more potently intimal thickening as in this case the biological action of NO is not circumscribed by immediate degradation upon exposure oxyhaemoglobin. Exposure of vascular tissue to NO delivery for a very short interval (5 minutes) has been proven in this and a previous study to exert long-lasting effects on tissue remodelling and patency [14]. These effects are of outmost clinical relevance in everyday treatment of arthrosclerosis.

Biochemical analysis of the arterial wall proteins actin, myosin and intermediate filaments was performed one week postoperatively, as histological examination is uncertain at this time point. The SMC cytoskeleton contains a high amount of IF but IF can be found in all types of cells. The function of IF in SMC is not established, but IF is increased in hypertrophy and hyperplasia [31]. The content of actin is not changed in smooth muscle in intimal hyperplasia when related to total tissue weight [32]. Since SMC adaption is associated with an increase in IF while the amount of actin is unchanged, we chose to use the IF/actin ratio as a measurement of PTCA induced adaption to arterial injury.

We found that SIN-1 treatment after PTCA affects the structural IF system in the SMC in a pronounced way. NO released by SIN-1 and applied to the injured area inhibits the increase of the IF/actin and IF/myosin ratios. On the contrary when the cGMP dependent NO-pathway is blocked by ODQ, IF/actin increase significantly, indicating a rapid turnover of IF proteins and cell growth after injury.

It cannot be excluded that the increased IF/actin and IF/myosin ratios seen after ODQ+SIN-1 treatment represents an increase in the number of non-SMC, activated at the time of injury. The present results suggest a possible role for NO in SMC remodeling in response to injury, as exposure to SIN-1 significantly reduced the IF/actin ratio, which directly correlated to the degree of neointimal formation at 8 weeks. Interestingly, a recent study has demonstrated that hematopoietic stem cells give rise to SMC after angioplasty [33]. If this is the case our results indicate that NO may influence stem cells.

In the heart, nitric oxide synthase (NOS) is found almost exclusively in vessels [30]. Practically none is found in the endocardium, valves or cardiac muscle fibers. Most NOS in the heart is in the form of the endothelial isoform (eNOS). Recently, it has been suggested that eNOS localized in the endothelial caveolae may act as a mechanosensor to couple NO release to long time hemodynamic changes that regulate extracellular matrix turnover, endothelial and SMC proliferation, migration and responsiveness to growth factors; events ultimately responsible for artery remodeling [8,24,25]. The inducible isoform (iNOS) is only found after injury [34]. In an attempt to establish the amount of NOS in the endothelium after PTCA treatment with SIN-1 or ODQ+SIN-1, NADPH diaphorase histochemistry of the arteries was performed. This histochemical reaction is sensitive to temperature and fixation-time. For this reason treated segments were always matched and compared with their distal untreated segment, which was harvested simultaneously. Although NADPH also marks other enzymes such as cytochrome P450 reductase, NADPH oxidase and non-specific phosphatases these enzymes are predominantly found in the liver [35,36]. This response was inhibited by SIN-1. We attributed this effect to the release of NO as addition of ODQ abolished the favorable result of SIN-1 application. Events modulating endothelial NOS appeared early after vascular injury as changes in NADPH diaphorase staining were present already at one week. The present findings suggest that NO down-regulates the expression of NOS in newly formed endothelial cells to normal levels and implicate a long lasting change in the mRNA expression of endothelial cells [37]. A plausible alternative explanation may be that the local delivery of SIN-1, leads to a greater degree of local endothelial loss, which is not recovered at later time-points as compared with NaCl treated animals. However, in this case one would expect decreased NADPH activity whenever SIN-1 is applied even in the presence of ODQ. Our results do not show decrease when ODQ is used, indicating that the SIN-1 effect is mediated through the cGMP dependent NO pathway, which requires functioning endothelium.

Porcine coronary arteries respond to injury with constrictive remodeling, elastic recoil and tissue proliferation, in a manner similar to human arteries [38,39]. Development of intimal hyperplasia is related to the degree of injury [20] and events at the onset [14]. There was no significant difference in the IEL fracture length inflicted to the arteries, but the arteries treated with SIN-1 had a 72% lower restenosis index, 71% less intimal hyperplasia compared to controls, and 26% larger residual lumen. This effect is apparently due to NO since it is abolished in the presence of ODQ.

## Conclusions

Events at the onset of injury are crucial to the process of neointimal formation. Intimal hyperplasia/restenosis is significantly reduced by application of SIN-1 immediately after PTCA. The mechanism underlying the effect of SIN-1 was shown to rely on NO release, which is inhibited by guanylyl cyclase, both in SMC as well as in endothelial cells.

## List of abbreviations used

PTCA: Percutaneous transluminal coronary angioplasty; LAD: left anterior coronary artery; LCX: Left circumflex coronary artery; SIN-1: 3-morpholino-sydnonimine; ODQ: 1 H-(1,2,4)oxadiazole(4,3-alpha)quinoxaline-1-one; IF: intermediate filaments; NOS: nitric oxide synthetase; cGMP: cyclic guanosine 3',5'-monophosphate; SMC: smooth muscle cells; I: intimal vascular layer; M: medial vascular layer; L: luminal area; IEL: internal elastic lamina; F: fracture length of the IEL; Injury index = F/IEL; IH: Intimal hyperplasia; (I/I+M)/(F/IEL: restenosis index; L/(L+I): residual lumen; eNOS: endothelial isoform; iNOS: inducible isoform

## Competing interests

The authors declare that they have no competing interests.

## Authors' contributions

JH and EZ drafted the primary study protocol. EE carried out the NADPH histochemistry. AA did all the electrophoretic examination. We all participated in the design of the study and performed the statistical analysis. US did all the pathology. VP did the entire anesthetic. All Authors conceived of the study, and participated in its design and coordination and helped to draft the manuscript. All authors read and approved the final manuscript.

## Pre-publication history

The pre-publication history for this paper can be accessed here:

http://www.biomedcentral.com/1471-2261/11/30/prepub

## References

[B1] Libby P Inflammation in atherosclerosis Nature 2002 420 6917 868 874 10.1038/nature01323 12490960

[B2] Pacher P Beckman JS Liaudet L Nitric oxide and peroxynitrite in health and disease Physiol Rev 2007 87 315 424 10.1152/physrev.00029.2006 17237348 PMC2248324

[B3] Sarkar K Sharma SK Sachdeva R Romeo F Mehta JL Coronary artery stenosis: vascular biology and emerging therapeutic strategies Expert Rev Cardiovasc Ther 2006 4 4 543 556 10.1586/14779072.4.4.543 16918273

[B4] Sarkar R Gordon D Stanley JC Webb RC Dual cell cycle-specific mechanism mediate the antimitogenic effects of nitric oxide in vascular smooth muscle cells Hypertens 1997 15 275 283 10.1097/00004872-199715030-00009 9468455

[B5] Münzel T Recent findings on nitrates: their action, bioactivation and development of tolerance Dtsch Wochensch 2008 133 44 2277 2282 10.1055/s-0028-1091272 18946854

[B6] Kim YM Bombeck CA Billiar TR Nitric oxide as a bifunctional regulator of apoptosis Circ.Res 1999 84 253 256 10024298 10.1161/01.res.84.3.253

[B7] Rabkin SW Nitric oxide-induced cell death in the heart. The role of autophagy Autophagy 2007 3 4 347 349 17438363 10.4161/auto.4054

[B8] Maxell AJ Mechanisms of dysfunction of the nitric oxide pathway in vascular disease Nitric Oxide 2002 6 2 101 124 10.1006/niox.2001.0394 11890735

[B9] Shiraki T Takamura A Oka T Saito D Effect of short-term administration of high dose L-Arginine on restenosis after percutaneous coronary angioplasty J Cardiol 2004 44 1 13 20 15334880

[B10] Yoon J Wu C-j Homme J Tuch TJ Wolff RG Topol EJ Lincoff AM Local delivery of nitrix oxide from a eluting stent to inhibit neointimal thickening in a porcine coronary injury model Yonsei Medical Journal 2002 43 2 242 251 11971219 10.3349/ymj.2002.43.2.242

[B11] Lablanche J-M Grollier G Lusson J-R Effect of direct nitric oxide donors Linsidomine and Molsidomine on angiographic restenosis after coronary balloon angioplasty. The ACCORD study Circulation 1997 95 1 83 89 8994421 10.1161/01.cir.95.1.83

[B12] Wohrle J Hoher M Nusser T Kocks M No effect of highly dosed nitric oxide donor molsidomine on the angiographic restenosis after percutaneous coronary angioplasty: a randomized, placebo controlled double-blind trial Can J Cardiol 2003 19 5 495 500 12717484

[B13] Baek SH Hrabie JA Keefer LK Hou D Fineberg N Rhoades R March KL Augmentation of intrapericardial nitric oxide level by a prolonged-release nitric oxide donor reduces luminal narrowing after porcine coronary angioplasty Circulation 2002 105 2779 2784 10.1161/01.CIR.0000017432.19415.3E 12057994

[B14] Harnek J Zoucas E Sjuve R Arner A Ekblad E Schou H Perez de Sà V Stenram U Local infusion of the NO donor SIN-1 after angioplasty reduces intimal hyperplasia in porcine coronary arteries Acta Radiol 2003 44 4 395 402 12846690 10.1080/j.1600-0455.2003.00079.x

[B15] Palmer RMJ Ferrige AG Moncada S Nitric oxide release accounts for the biological activity of endothelium-derived relaxing factor Nature 1987 327 524 526 10.1038/327524a0 3495737

[B16] Groves PH Lewis MJ Cheadle HA Penny WJ SIN-1 reduces platelet adhesion and platelet thrombus formation in a porcine model of balloon angioplasty Circulation 1993 87 590 597 8425303 10.1161/01.cir.87.2.590

[B17] Furlong B Henderson AM Lewis MJ Smith JA Endothelium-derived relaxing factor inhibits in vitro platelet aggregation Br. J. Pharmcol 1987 90 4 687 692 10.1111/j.1476-5381.1987.tb11221.x PMC1917198 3495310

[B18] Malmqvist U Arner A Isoform distribution and tissue content of contractile and cytoskeletal proteins in hypertrophied smooth muscle from rat portal vein Circ. Res 1990 66 3 832 845 2306809 10.1161/01.res.66.3.832

[B19] Hope B Michael GJ Knigge KM Vincent SR Neuronal NADPH diaphorase is a nitric oxide synthase Proc Natl Acad Sci USA 1991 88 2811 2814 10.1073/pnas.88.7.2811 1707173 PMC51329

[B20] Nugent HM Rogers C Edelman ER Endothelial implants inhibit intimal hyperplasia after porcine angioplasty Circ Res 1999 84 384 391 10066672 10.1161/01.res.84.4.384

[B21] Bonan R Paiement P Scortichini D Cloutier MJ Leung TK Coronary restenosis: Evaluation of a restenosis injury index in a swine model Am Heart J 1993 126 6 1334 1340 10.1016/0002-8703(93)90531-D 8249790

[B22] Lee KW Norell MS Management of "no-reflow" complicating reperfusion therapy Acute Card Care 2008 10 1 5 14 10.1080/17482940701744318 18449813

[B23] Pilz RB Catsteel DE Regulation of gene expression by cGMP Circ Res 2003 93 1034 1046 10.1161/01.RES.0000103311.52853.48 14645134

[B24] Rudic RD Shesely EG Nobuyo M Maeda N Smithies O Segal SS Sessa WC Direct evidence for the importance of endothelium derived Nitric Oxide in vascular remodeling J.Clin.Invest 1998 101 4 731 736 10.1172/JCI1699 9466966 PMC508619

[B25] Gratton JP Bernatchez P Sessa WC Caveola and calveolins in the cardiovascular system Circ.Res 2004 94 1408 1417 10.1161/01.RES.0000129178.56294.17 15192036

[B26] Shen YH Wang XL Wilken DE Nitric oxide induces and inhibits apoptosis through different pathways FEBS Lett 1998 433 1-2 125 131 10.1016/S0014-5793(98)00844-8 9738946

[B27] Brüne B von Knethen A Sandau KB Nitric oxide and its role in apoptosis Eur.J.Pharmacol 1998 351 261 272 10.1016/S0014-2999(98)00274-X 9721017

[B28] Beckman JS Koppenol WH Nitric oxide, superoxide and peroxynitrite: The good, the bad and the ugly Am.J.Physiol 1996 271 C1424 1437 8944624 10.1152/ajpcell.1996.271.5.C1424

[B29] Radi R Beckman JS Bush KM Freeman BA Peroxynitrite oxidation of sulfhydryls. The cytotoxic potential of superoxide and nitric oxide J.Biol.Chem 1991 266 7 4244 4250 1847917

[B30] Kaji T Kaieda I Hisatsune T Kaminogawa S 3-Morpholinosydnonimine hydrochloride induces p53-dependent apoptosis in murine primary neural cells: A critical role for p21^ras^-MAPK-p19^ARF ^pathway Nitric Oxide 2002 6 2 125 134 10.1006/niox.2001.0389 11890736

[B31] Kocher O Gabbiani G Cytoskeletal features of normal and atheromatous human arterial smooth muscle cells Hum Pathol 1986 17 9 875 880 10.1016/S0046-8177(86)80637-2 3759072

[B32] Gabbiani G Kocher O Bloom WS Vandekerckhove J Weber K Actin expression in smooth muscle cells of rat aortic intimal thickening, human atheromatous plaque, and cultured rat aortic media J Clin Invest 1984 73 1 148 152 10.1172/JCI111185 6690475 PMC424985

[B33] Sata M Saiura A Kunisato A Tojo A Okada S Tokuhisa T Hirai H Makuuchi M Hirata Y Nagai R Hematopoietic stem cells differentiate into vascular cells that participate in the pathogenesis of atherosclerosis Nat Med 2002 8 4 403 409 10.1038/nm0402-403 11927948

[B34] Ursell PC Mayes M Anatomic distribution of nitric oxide synthetase in the heart Int J Cardiol 1995 50 3 217 223 10.1016/0167-5273(95)02380-F 8537144

[B35] Persson K Poljakovic M Johansson K Larsson B Morphological and biochemical investigation of nitric oxide synthetase and related enzymes in the rat and pig urothelium J Histochem Cytochem 1999 47 6 739 750 10.1177/002215549904700603 10330450

[B36] Shi Y Niculescu R Wang D Patel S Davenpeck KL Zalewski A Increased NAD(P)H oxidase and reactive species in coronary arteries after balloon injury Arterioscler Tromb Vasc Biol 2001 21 739 745 10.1161/01.ATV.21.5.739 11348868

[B37] Brüne B Sandau K Knethen A Apoptotic cell death and nitric oxide: activating and antagonistic transducing pathways Biochemistry (Mosc) 1998 63 7 817 825 9721334

[B38] Nobuyoshi M Kimura T Ohishi H Horiuchi H Nosaka H Hamasaki N Yokoi H Kim K Restenosis after percutaneous transluminal coronary angiography: pathologic observations in 20 patients J Am Coll Cardiol 1991 17 2 433 439 10.1016/S0735-1097(10)80111-1 1991900

[B39] Mintz GS Popma JJ Hong MK Pichard AD Kent KM Satler LF Leon MB Intravascular ultrasound to discern device-specific effects and mechanisms of restenosis Am J Cardiol 1996 78 3A 18 22 8751842 10.1016/s0002-9149(96)00493-6

